# Autogenous tooth transplantation of canines: a prospective clinical study on the influence of extraoral storage time-guided adjunctive antibiotic therapy and patient-related risk factors affecting success, survival, and prognosis after two years of follow-up

**DOI:** 10.1186/s12903-026-07697-w

**Published:** 2026-01-31

**Authors:** Sebastian Meinzer, Dirk Nolte, Karin Christine Huth

**Affiliations:** 1 Department of Conservative Dentistry, Periodontology and Digital Dentistry, LMU University Hospital, LMU Munich, Goethestraße 70, 80336 Munich, Germany; 2 Practice Clinic for Oral and Maxillofacial Surgery, Sauerbruchstraße 48, 81377 Munich, Germany; 3 TUM School of Medicine and Health, University Hospital Rechts Der Isar, Technische Universität München (TUM), Ismaninger Straße 22, 81675 Munich, Germany; 4 Department of Oral and Maxillofacial Surgery, LMU University Hospital, LMU Munich, Lindwurmstraße 2a, 80337 Munich, Germany

**Keywords:** Autogenous tooth transplantation, Canine, Prospective study, Adjunctive antibiotic therapy, Risk factors, Two-year follow-up, Success, Survival` prognostic estimate

## Abstract

**Background:**

The aim of this study was to evaluate the influence of adjunctive antibiotic therapy and patient-related factors on the success, survival, and prognosis of autogenous canine transplants over a two-year period.

**Methods:**

Sixty-seven patients (aged 11–37 years) underwent canine autotransplantation. According to extraoral storage time (EST: 0–3, 4–6, or 7–15 min), patients received either (1) no antibiotics, (2) a single intraoperative intravenous dose of doxycycline, or (3) the same dose followed by a five-day oral regimen. Because allocation of systemic adjunctive antibiotic therapy was EST-based, their effects could not be assessed independently. Clinical and radiographic evaluations were performed. Primary outcomes were survival and success. The secondary outcome was a composite prognostic estimate (percentage of criteria fulfilled). Associations were analysed using linear regression.

**Results:**

After two years, the success rate was 90% (95% CI: 82–97%), survival was 100%, and the mean prognostic estimate was 86% (95% CI: 82–89%). The adjunctive antibiotic therapy regimen had no significant effect. Success decreased with smoking (β = -8.03; p = 0.025). The prognostic estimate declined with increasing age (β = -1.30 per year), closed apex (β = -11.84), ankylosis (β = -13.80), orthodontic extrusion (β = -12.04) and its duration (β = -0.49 per month), and smoking (β = -14.15) (all *p* < 0.05).

**Conclusions:**

Routine adjunctive antibiotic therapy did not improve outcomes when allocated according to EST, supporting antibiotic stewardship. Prognosis was mainly influenced by patient-specific factors, highlighting the importance of individualised risk assessment in canine autotransplantation.

**Trial registration:**

DRKS00034011 (German Clinical Trials Register and WHO International Clinical Trials Registry Platform); registered retrospectively on 4 April 2024. https://trialsearch.who.int/Trial2.aspx?TrialID=DRKS00034011.

**Supplementary Information:**

The online version contains supplementary material available at 10.1186/s12903-026-07697-w.

## Background

Impaction and retention of permanent canines are common eruption disorders, affecting about 2% of the population [1]. They result from the long, curved eruption pathway of canines and may cause root resorption, displacement, or functional impairment [2–7]. The standard management is orthodontic extrusion after surgical exposure [8, 9]. When orthodontic treatment is not feasible or fails, autogenous tooth transplantation offers a biological alternative to extraction or prosthetic replacement [10–12]. Indications include severe displacement, prolonged retention, or failed exposure and bracketing attempts [13–15]. Reported survival rates exceed 90% after several years [16, 17].

Given its surgical nature, canine autotransplantation is often accompanied by adjunctive antibiotic therapy, although evidence-based guidelines are lacking and current findings are inconsistent [18, 19]. This is clinically relevant, particularly in adolescents, who are most frequently affected and more susceptible to antibiotic side effects [20]. In addition, indiscriminate antibiotic use promotes antimicrobial resistance [21, 22]. Doxycycline, a tetracycline derivative with antiresorptive effects, is frequently used, yet its benefit remains unproven [23, 24].

This prospective clinical study investigates the influence of adjunctive antibiotic therapy and patient-related factors on survival, success, and long-term prognosis of autotransplanted canines over two years. We hypothesized that routine adjunctive antibiotic therapy does not improve outcomes and that patient-related risk factors have a greater impact. Preliminary findings from our earlier report showed no benefit of antibiotics for short-term healing, while age, smoking, ankylosis, and orthodontic extrusion were associated with compromised early outcomes [25].

## Methods

### Trial design and participants

This prospective clinical trial was conducted between 2021 and 2023 with follow-ups until 2025 at a private oral and maxillofacial surgery clinic in Munich in cooperation with the Department of Conservative Dentistry, Periodontology and Digital Dentistry, University Hospital, LMU Munich. Ethical approval was obtained from the LMU ethics committee (Project No. 20–0903), and the study was registered in the German Clinical Trials Register (DRKS00034011). Reporting followed CONSORT and TREND guidelines [26, 27]. Sixty-seven patients (11–37 years) requiring autogenous canine transplantation were enrolled. No formal sample-size calculation was performed because of the exploratory design.

#### Inclusion criteria

Inclusion criteria comprised an indication for autotransplantation due to confirmed retention or impaction after failed orthodontic alignment attempts [28, 29], informed consent (patient and legal guardian if underage), minimum age 11 years (consistent with doxycycline approval [30]), and satisfactory oral hygiene (plaque, calculus, and gingival index ≤ 1).

#### Exclusion criteria

Exclusion criteria included systemic or metabolic disorders affecting bone metabolism, immunodeficiency, malignancy, coagulopathies, medication interfering with bone turnover (corticosteroids, bisphosphonates, denosumab), psychomotor impairment, or substance abuse. Patients smoking > 20 cigarettes/day or unable to comply with follow-up were also excluded.

### Allocation

Participants were assigned to three parallel groups according to intraoperative extraoral storage time (EST) of the donor tooth: Group 1 = 0–3 min, Group 2 = 4–6 min, Group 3 = 7–15 min. Randomisation was not permitted by the ethics board because of the participants’ age and ethical constraints. Allocation based on EST was chosen due to its clinical relevance for periodontal ligament integrity [31, 32]. Because group allocation was based on intraoperative EST, EST and systemic adjunctive antibiotic therapy were inherently linked; therefore, their effects could not be assessed independently in this study design. All surgeries were performed by a single experienced oral and maxillofacial surgeon. When two canines were transplanted, the tooth for follow-up was randomly selected by an assistant using sealed envelopes.

### Interventions and follow-ups

After full medical and dental documentation and written consent, surgery was scheduled. Where feasible, other necessary procedures (e.g., third molar extraction) were combined in the same session. All operations were performed under general anaesthesia (propofol and transnasal intubation).

A mucoperiosteal flap was elevated, and the recipient site was prepared by osteotomy. The donor tooth was extracted carefully to preserve the root surface and placed into the prepared socket. During the extraoral phase, the tooth was stored in a sterile solution containing dexamethasone (Dex-ratiopharm® injection; 4 mg/ml; ratiopharm GmbH, Ulm, Germany), doxycycline (Doxycyclin-ratiopharm® SF injection; 100 mg/5 ml; ratiopharm GmbH, Ulm, Germany), and saline (0.9% NaCl injection; 10 ml; B. Braun Melsungen AG, Melsungen, Germany) [33, 34]. The transplant was stabilized with a saliva-proof adhesive splint, and soft tissue was closed with interrupted sutures. This extraoral storage protocol was applied uniformly in all cases, irrespective of systemic adjunctive antibiotic therapy allocation.

Follow-ups were performed on days 1, 7, and 21 (postoperative wound control, suture and splint removal), then at 3, 6, 9, 12, and 24 months, including clinical and radiographic assessment. Early signs of pathology prompted appropriate intervention such as endodontic therapy or modification of orthodontic treatment. Orthodontic extrusion or bracketing started in week 4 when required; if no orthodontic intervention was needed, the transplant was left unsplinted after four weeks once stability was confirmed. Analgesia consisted of ibuprofen (200–600 mg as needed).

Group-specific adjunctive antibiotic therapy protocols were applied: Group 1 received no systemic adjunctive antibiotic therapy; Group 2 received a single intravenous dose of doxycycline (100 mg) intraoperatively; and Group 3 was given the same intravenous dose followed by oral doxycycline (50–100 mg according to body weight) administered twice daily for five days. Postoperative infection was treated through early removal of sutures, saline irrigation, and, if necessary, oral administration of amoxicillin/clavulanic acid (500/125 mg or 875/125 mg depending on body weight) twice daily for five days. All follow-ups were conducted by one calibrated dentist.

### Complication management during follow-ups

Main complications were early ankylosis and apical periodontitis. In such cases, orthodontic force application was halted to prevent root resorption [35, 36].

Endodontic therapy was initiated upon radiographic evidence of apical osteolysis or resorption [37]. The standardised protocol included access preparation, pulp extirpation, canal instrumentation, irrigation with sodium hypochlorite and EDTA, calcium-hydroxide dressing, and temporary sealing with composite. The procedure was repeated if necessary until radiographic healing occurred. In selected orthodontic cases, calcium-hydroxide dressing was maintained longer to control resorption [38–40]. Once radiographic healing was confirmed and orthodontic treatment had been completed or brackets had been removed, the patient was referred to endodontic specialists for definitive obturation.

### Outcomes

Patient, surgical, and follow-up data were recorded. After one year, participants completed an anonymous satisfaction survey (grades 1–6) evaluating expectations, aesthetics, discomfort, and overall success; a mean satisfaction score was calculated.

The primary outcome was the 2-year success rate, defined as the percentage of transplants fulfilling all success criteria. A single unmet criterion classified the case as failure.

Clinical success required mobility grade 0 [41], mean probing depth < 3.5 mm [42], and absence of symptoms [17]. Radiographic success required no periapical radiolucency or resorption [43].

The secondary outcome was the survival rate (tooth in situ after 2 years) [44]. Additionally, a prognostic estimate was calculated, representing the percentage of long-term prognostic criteria fulfilled (Table 2). Clinical prognostic criteria included absence of ankylosis [45], negative percussion [46], positive pulp vitality [47], gingival recession < 1 mm [48], adequate proximal contacts [49], and static/dynamic occlusion [50]. Furthermore, proper horizontal alignment within the dental arch without rotation or vestibular, palatal, or lingual inclination was included [51]. Radiographic criteria comprised no need for endodontic treatment [52], maintenance or gain of root length [53] and crestal bone level [54], pulpal obliteration [55], and absence of caries on the transplant or adjacent teeth [56].

Radiographic evaluation used standardised periapical radiographs analysed with digital imaging software (Sidexis 4, Version 4.4, Dentsply Sirona, Bensheim, Hesse, Germany; 3D Slicer, Version 5.8.1, The Slicer Community, Boston, MA, USA). Root length was measured from the apex to the midpoint of the cemento-enamel junction, and crestal bone level from the CEJ to the alveolar crest [57, 58].

Independent variables included adjunctive antibiotic therapy regimen, sex, age, smoking, apex condition, preoperative ankylosis, displacement severity, jaw location, orthodontic extrusion and duration, EST, and intraoperative complications. Ankylosis was defined radiographically (loss of periodontal ligament space or replacement resorption) and clinically (metallic percussion sound) [59]. Displacement severity followed established classification systems [60].

An overview of the independent and dependent variables is provided in Table 1, referring to the same cohort as reported in the publication on initial healing [25]. A summary of the success criteria and prognostic criteria is presented in Table 2.

**Table 1:** Independent and dependent variables of the same cohort as reported in the publication on initial healing [25]. Shown are the categories or value ranges and the corresponding data for the overall sample as well as for each group

Subtype	Variable	Range/Categories	All Groups	Group 1	Group 2	Group 3
**Independent**	Group	1, 2, 3	67 participants (100%)	24 participants (36%)	21 participants (31%)	22 participants (33%)
	Sex	female, male	43 females (64%) 24 males (36%)	12 females (50%) 12 males (50%)	14 females (67%) 7 males (33%)	17 females (77%) 5 males (23%)
	Age	11–37 years	median of 14 years (+/− 4.9)	median of 12 years (+/− 5.2)	median of 15 years (+/− 5.6)	median of 14.5 years (+/− 3.1)
	Nicotine abuse	no, yes	*n* = 6 with nicotine abuse (9%) *n* = 61 without nicotine abuse (91%)	*n* = 1 with nicotine abuse (4%) *n* = 23 without nicotine abuse (96%)	*n* = 3 with nicotine abuse (14%) *n* = 18 without nicotine abuse (86%)	*n* = 2 with nicotine abuse (9%) *n* = 20 without nicotine abuse (91%)
	Condition of tooth apex	open, closed	*n* = 19 with open apex (28%) *n* = 48 with closed apex (72%)	*n* = 13 with open apex (54%) *n* = 11 with closed apex (46%)	*n* = 3 with open apex (14%) *n* = 18 with closed apex (86%)	*n* = 3 with open apex (14%) *n* = 19 with closed apex (86%)
	Preoperative ankylosis	no, yes	*n* = 51 without ankylosis (76%) *n* = 16 with ankylosis (24%)	*n* = 18 without ankylosis (75%) *n* = 6 with ankylosis (25%)	*n* = 14 without ankylosis (67%) *n* = 7 with ankylosis (33%)	*n* = 19 without ankylosis (86%) *n* = 3 with ankylosis (14%)
	Severity of displacement	mild, moderate, severe	*n* = 14 with mild displacement (21%) *n* = 21 with moderate displacement (31%) *n* = 32 with severe displacement (48%)	*n* = 5 with mild displacement (21%) *n* = 8 with moderate displacement (33%) *n* = 11 with severe displacement (46%)	*n* = 4 with mild displacement (19%) *n* = 6 with moderate displacement (29%) *n* = 11 with severe displacement (52%)	*n* = 5 with mild displacement (23%) *n* = 7 with moderate displacement (32%) *n* = 10 with severe displacement (45%)
	Jaw	maxilla, mandible	*n* = 51 in the maxilla (76%) *n* = 16 in the mandible (24%)	*n* = 18 in the maxilla (75%) *n* = 6 in the mandible (25%)	*n* = 15 in the maxilla (71%) *n* = 6 in the mandible (29%)	*n* = 18 in the maxilla (82%) *n* = 4 in the mandible (18%)
	Preoperative orthodontic extrusion on the transplant	no, yes	*n* = 51 without extrusion (76%) *n* = 16 with extrusion (24%)	*n* = 19 without extrusion (79%) *n* = 5 with extrusion (21%)	*n* = 13 without extrusion (62%) *n* = 8 with extrusion (38%)	*n* = 19 without extrusion (86%) *n* = 3 with extrusion (14%)
	Duration of orthodontic extrusion	0–99 months	mean of 5 months (± 10)	mean of 4 months (± 8)	mean of 8 months (± 12)	mean of 2 months (± 8)
	Extraoral storage time (EST)	0–15 min	median of 4 min (+/− 3.0)	median of 2 min (+/− 0.6)	median of 4 min (+/− 0.7)	median of 8 min (+/− 1.7)
	Intraoperative complication	no, yes	*n* = 54 without complication (81%) *n* = 13 with complication (19%)	*n* = 20 without complication (83%) *n* = 4 with complication (17%)	*n* = 14 without complication (67%) *n* = 7 with complication (33%)	*n* = 20 without complication (91%) *n* = 2 with complication (9%)
**Dependent**	Survival Rate	0–100%	mean of 100%	mean of 100%	mean of 100%	mean of 100%
	Success Rate	0–100%	mean of 90% (95% CI: 82–97%)	mean of 96% (95% CI: 87–104%)	mean of 81% (95% CI: 63–99%)	mean of 91% (95% CI: 78–104%)
	Prognostic Estimate	0–100%	mean of 86% (95% CI: 82–89%)	mean of 88% (95% CI: 82–94%)	mean of 83% (95% CI: 77–90%)	mean of 86% (95% CI: 80–92%)

**Table 2:** Success criteria and prognostic criteria. Shown are the categories and the corresponding data for the overall sample as well as for each group

Classification	Subtype	Variable	Range/Categories	All Groups	Group 1	Group 2	Group 3
**Success Criteria**	**Clinical**	Mobility	no, yes	*n* = 67 without mobility (100%)	*n* = 24 without mobility (100%)	*n* = 21 without mobility (100%)	*n* = 22 without mobility (100%)
		Probing depths	no, yes	*n* = 66 with physiological PD (99%) *n* = 1 without physiological PD (1%)	*n* = 24 with physiological PD (100%)	*n* = 20 with physiological PD (95%) *n* = 1 without physiological PD (5%)	*n* = 22 with physiological PD (100%)
		Pain	no, yes	*n* = 67 without pain (100%)	*n* = 24 without pain (100%)	*n* = 21 without pain (100%)	*n* = 22 without pain (100%)
	**Radiological**	Apical status	no, yes	*n* = 65 with physiological AS (97%) *n* = 2 without physiological AS (3%)	*n* = 23 with physiological AS (96%) *n* = 1 without physiological AS (4%)	*n* = 21 with physiological AS (100%)	*n* = 21 with physiological AS (95%) *n* = 1 without physiological AS (5%)
		Resorption	no, yes	*n* = 61 without resorption (91%) *n* = 6 with resorption (9%)	*n* = 23 without resorption (96%) *n* = 1 with resorption (4%)	*n* = 18 without resorption (86%) *n* = 3 with resorption (14%)	*n* = 20 without resorption (91%) *n* = 2 with resorption (9%)
**Prognostic Criteria**	**Clinical**	Ankylosis	no, yes	*n* = 49 without ankylosis (73%) *n* = 18 with ankylosis (27%)	*n* = 19 without ankylosis (79%) *n* = 5 with ankylosis (21%)	*n* = 13 without ankylosis (62%) *n* = 8 with ankylosis (38%)	*n* = 17 without ankylosis (77%) *n* = 5 with ankylosis (23%)
		Percussion	no, yes	*n* = 67 without percussion (100%)	*n* = 24 without percussion (100%)	*n* = 21 without percussion (100%)	*n* = 22 without percussion (100%)
		Vitality	no, yes	*n* = 37 with vitality (55%) *n* = 30 without vitality (45%)	*n* = 15 with vitality (63%) *n* = 9 without vitality (37%)	*n* = 8 with vitality (38%) *n* = 13 without vitality (62%)	*n* = 14 with vitality (64%) *n* = 8 without vitality (36%)
		Gingival recession	no, yes	*n* = 63 with physiological GR (94%) *n* = 4 without physiological GR (6%)	*n* = 22 with physiological GR (92%) *n* = 2 without physiological GR (8%)	*n* = 21 with physiological GR (100%)	*n* = 20 with physiological GR (91%) *n* = 2 without physiological GR (9%)
		Proximal contacts	no, yes	*n* = 63 with sufficient PC (94%) *n* = 4 without sufficient PC (6%)	*n* = 23 with sufficient PC (96%) *n* = 1 without sufficient PC (4%)	*n* = 20 with sufficient PC (95%) *n* = 1 without sufficient PC (5%)	*n* = 20 with sufficient PC (91%) *n* = 2 without sufficient PC (9%)
		Static occlusion	no, yes	*n* = 61 with sufficient SO (91%) *n* = 6 without sufficient SO (9%)	*n* = 21 with sufficient SO (88%) *n* = 3 without sufficient SO (12%)	*n* = 20 with sufficient SO (95%) *n* = 1 without sufficient SO (5%)	*n* = 20 with sufficient SO (91%) *n* = 2 without sufficient SO (9%)
		Dynamic occlusion	no, yes	*n* = 61 with sufficient DO (91%) *n* = 6 without sufficient DO (9%)	*n* = 21 with sufficient DO (88%) *n* = 3 without sufficient DO (12%)	*n* = 20 with sufficient DO (95%) *n* = 1 without sufficient DO (5%)	*n* = 20 with sufficient DO (91%) *n* = 2 without sufficient DO (9%)
		Horizontal position	no, yes	*n* = 63 with sufficient position (94%) *n* = 4 without sufficient position (6%)	*n* = 23 with sufficient position (96%) *n* = 1 without sufficient position (4%)	*n* = 19 with sufficient position (90%) *n* = 2 without sufficient position (10%)	*n* = 21 with sufficient position (95%) *n* = 1 without sufficient position (5%)
	**Radiological**	Root canal treatment	no, yes	*n* = 46 without RCT (69%) *n* = 21 with RCT (31%)	*n* = 18 without RCT (75%) *n* = 6 with RCT (25%)	*n* = 12 without RCT (57%) *n* = 9 with RCT (43%)	*n* = 16 without RCT (73%) *n* = 6 with RCT (27%)
		Root length	no, yes	*n* = 66 with physiological RL (99%) *n* = 1 without physiological RL (1%)	*n* = 24 with physiological RL (100%)	*n* = 21 with physiological RL (100%)	*n* = 21 with physiological RL (95%) *n* = 1 without physiological RL (5%)
		Bone level	no, yes	*n* = 61 with physiological BL (91%) *n* = 6 without physiological BL (9%)	*n* = 23 with physiological BL (96%) *n* = 1 without physiological BL (4%)	*n* = 19 with physiological BL (90%) *n* = 2 without physiological BL (10%)	*n* = 19 with physiological BL (86%) *n* = 3 without physiological BL (14%)
		Pulp obliteration	no, yes	*n* = 33 with obliteration (49%) *n* = 34 without obliteration (51%)	*n* = 14 with obliteration (58%) *n* = 10 without obliteration (42%)	*n* = 8 with obliteration (38%) *n* = 13 without obliteration (62%)	*n* = 11 with obliteration (50%) *n* = 11 without obliteration (50%)
		Caries on the transplant	no, yes	*n* = 66 without caries (99%) *n* = 1 with caries (1%)	*n* = 24 without caries (100%)	*n* = 21 without caries (100%)	*n* = 21 without caries (95%) *n* = 1 with caries (5%)
		Proximal caries on adjacent teeth	no, yes	*n* = 66 without adjacent caries (99%) *n* = 1 with adjacent caries (1%)	*n* = 23 without adjacent caries (96%) *n* = 1 with adjacent caries (4%)	*n* = 21 without adjacent caries (100%)	*n* = 22 without adjacent caries (100%)

### Statistical analysis

Data were compiled in Microsoft Excel (Version 16.16.27, Microsoft Corporation, Redmond, WA, USA) and analysed in RStudio (Open Source Edition, Version 2024.09.1, Posit PBC, Boston, MA, USA). Descriptive statistics included mean, standard deviation, 95% confidence intervals, range, and median. Normality was tested by Shapiro–Wilk. Associations between independent and dependent variables were explored by univariate linear regression (α = 0.05) [61]. Effect size was quantified by partial eta-squared (ηp^2^) [62]; ηp^2^ = 0.01, 0.06, and ≥ 0.14 indicated small, medium, and large effects, respectively.

Intra-rater reliability was assessed on 10 randomly selected cases (15% of the cohort) re-evaluated after four weeks under identical conditions. Agreement rates > 80% were considered good and ≥ 90% very good [63]. The Intraclass Correlation Coefficient (ICC) was calculated: < 0.5 = poor, 0.5–0.75 = moderate, 0.75–0.9 = good, > 0.9 = excellent [64]. Intra-rater reliability analysis was conducted for radiographic variables requiring quantitative measurement, namely root length and crestal bone level. Other radiographic parameters were assessed as categorical outcomes based on predefined diagnostic criteria and standardised evaluation protocols and were therefore not subjected to separate reliability testing.

Survival and success rates were calculated using the crude rate method, according to the following formula [65]:

$$\begin{aligned} &\text{Survival or Success Rate}\\&=\frac{\text{Number of surviving or successful cases}}{\text{Total number of cases}}\times100\% \end{aligned}$$

The prognostic estimate was calculated as the mean of all individual prognostic estimates of the transplants, according to the same method, with the following adapted formula:

$$\begin{aligned} &\text{Prognostic Estimate}\\&=\frac{\text{Number of fulfilled criteria of an individual transplant}}{\text{Maximum possible criteria of the individual transplant}}\times100\% \end{aligned}$$

Patient satisfaction was likewise determined as the mean of all individual ratings per patient:

$$\begin{aligned} &\text{Patient Satisfaction}\\&=\frac{\text{Sum of individual school grades across all five categories}}{\text{Total number of categ}{\mathrm{ories}}} \end{aligned}$$

## Results

### Patient data

**Fig. 1:**
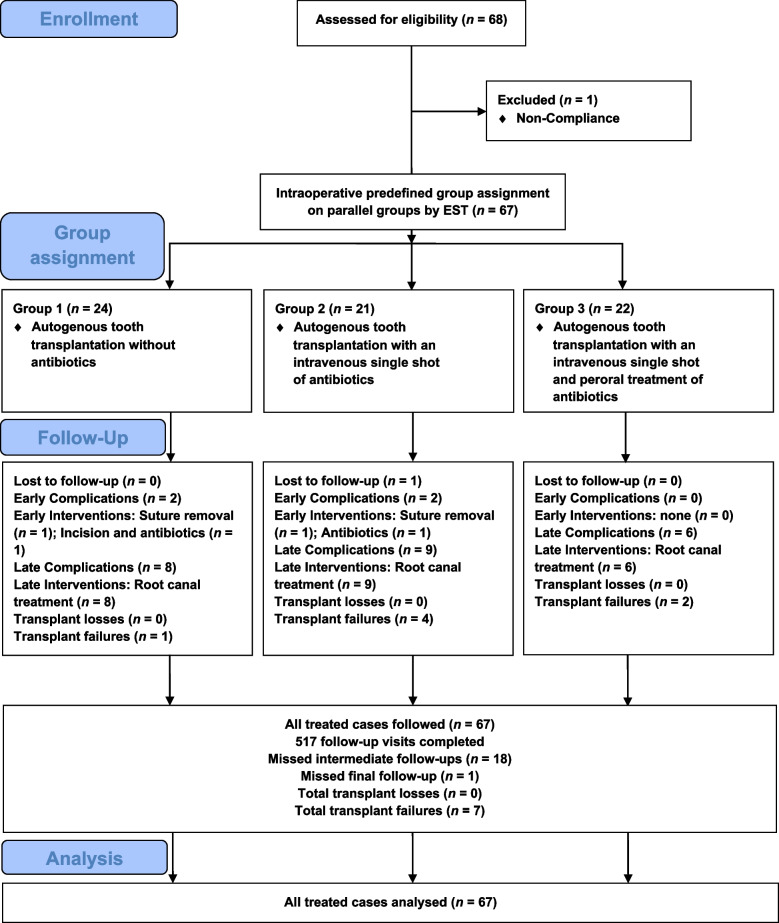
Flow of participants

As illustrated in Fig. 1, a total of *n* = 67 patients were included (43 females, 64%; 24 males, 36%), aged 11–37 years (mean 15.6 ± 5.8; median 14). Group 1 (*n* = 24) had an extraoral storage time (EST) of 0–3 min and received no antibiotics; Group 2 (*n* = 21) had EST 4–6 min and received a single intraoperative intravenous doxycycline dose; Group 3 (*n* = 22) had EST 7–15 min and received the same dose plus oral doxycycline for 5 days. Six participants (9%) reported nicotine use (10–20 cigarettes/day). Minor medical conditions were present in 17 patients (25%), and 16% took regular medication.

Across the study period, 517 follow-ups were performed. Eighteen interim appointments were missed, and one participant was lost to the final follow-up but included in the analysis according to the intention-to-treat principle [66].

### Operation data

As shown in Table 1 [25], 28% of teeth had open apices and 72% closed; preoperative ankylosis occurred in 24%. Displacement severity was mild in 21%, moderate in 31%, and severe in 48%. Transplants were placed in the maxilla in 76% and in the mandible in 24%. Mean EST was 4.99 ± 2.98 min (range 1–12). At surgery, 93% of patients were in active orthodontic treatment, and 24% had previous unsuccessful extrusion (mean 19.7 months). Intraoperative complications occurred in 19%; postoperative complications in 3% within 3 weeks. Adverse drug reactions were reported by 9% during week 1 only.

### Follow-up data

An overview of all data is presented in Table 2. After 2 years, all transplanted teeth (*n* = 67; 100%) fulfilled survival criteria. Clinically, all transplants were immobile and asymptomatic; one (1%) exceeded the probing-depth threshold. Radiographically, two cases (3%) showed persistent apical osteolysis and six (9%) root resorption, resulting in seven failures (10%) overall.

According to clinical prognostic criteria, ankylosis was found in 18 cases (27%). All transplants (100%) had negative percussion response, while 30 (45%) lacked pulp vitality. Gingival recession ≥ 1 mm occurred in 4 cases (6%); insufficient proximal contacts in 4 (6%); occlusal deficiencies in 6 (9%); and horizontal malposition in 4 (6%).

Among radiological prognostic criteria, 21 teeth (31%) required endodontic therapy. Root length was maintained or increased in 66 (99%) cases; one (1%) with closed apex showed minor apical resorption. Crestal bone level was preserved or improved in 61 (91%), while 6 (9%) showed localized vertical loss. Pulp canal obliteration was observed in 33 cases (49%). Caries on transplants and adjacent teeth each occurred in 1% of cases.

Intra-rater reliability was excellent, with 90% agreement for root length and 100% for crestal bone level (ICC = 0.93 and 0.99, respectively).

Quantitative radiographic analysis demonstrated a mean root length increase of 0.58 ± 0.51 mm in teeth with open apices (*n* = 19). Group-specific means were 0.62 ± 0.61 mm (Group 1), 0.33 ± 0.29 mm (Group 2), and 0.62 ± 0.25 mm (Group 3). Teeth with closed apices (*n* = 48) showed minimal change (0.02 ± 0.18 mm). Mean bone height gain was 0.42 ± 0.65 mm overall, with subgroup values of 0.46 ± 0.51 mm, 0.48 ± 0.86 mm, and 0.32 ± 0.59 mm for Groups 1–3, respectively.

### Patient satisfaction

After 1 year, satisfaction ratings were high: expectations 1.2, perceived success 1.2, aesthetics 1.3, postoperative discomfort 2.4, and current discomfort 1.0, yielding an overall satisfaction of 1.4 (“very good”). Ninety-five percent of participants would undergo autotransplantation again, and 100% would recommend it to others.

### Primary and secondary outcomes

The primary outcome, the success rate, was 90% (95% CI 82–97%), with group-specific rates of 96% (87–104%), 81% (63–99%), and 91% (78–104%) for Groups 1–3, respectively.

Secondary outcomes were also favourable. The overall survival rate was 100%, consistent across all groups. The mean prognostic estimate was 86% (95% CI 82–89%): Group 1 = 88% (82–94%), Group 2 = 83% (77–90%), and Group 3 = 86% (80–92%).

**Fig. 2:**
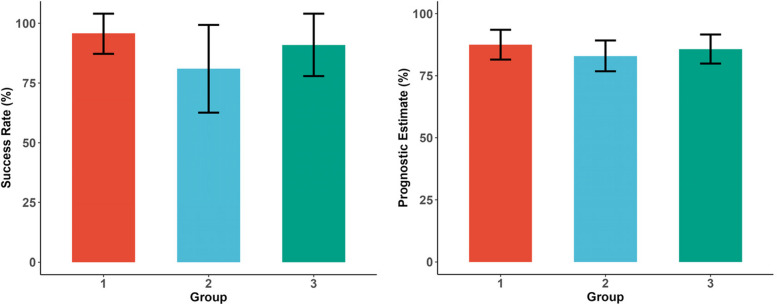
Success rates and prognostic estimates per group. Error bars represent 95% confidence intervals

As shown in Fig. 2, the success rate and prognostic estimate, including the corresponding confidence intervals, are illustrated for each group.

### Factors influencing the success rate

Linear regression revealed no significant effect of adjunctive antibiotic therapy group, sex, age, apex condition, displacement severity, jaw location, ankylosis, orthodontic extrusion or its duration, EST, or intraoperative complications (all *p* > 0.05). Smoking was the only significant factor (β = –8.03; *p* = 0.025; ηP^2^ = 0.075), explaining approximately 6% of variance (adjusted R^2^ = 0.061).

**Fig. 3:**
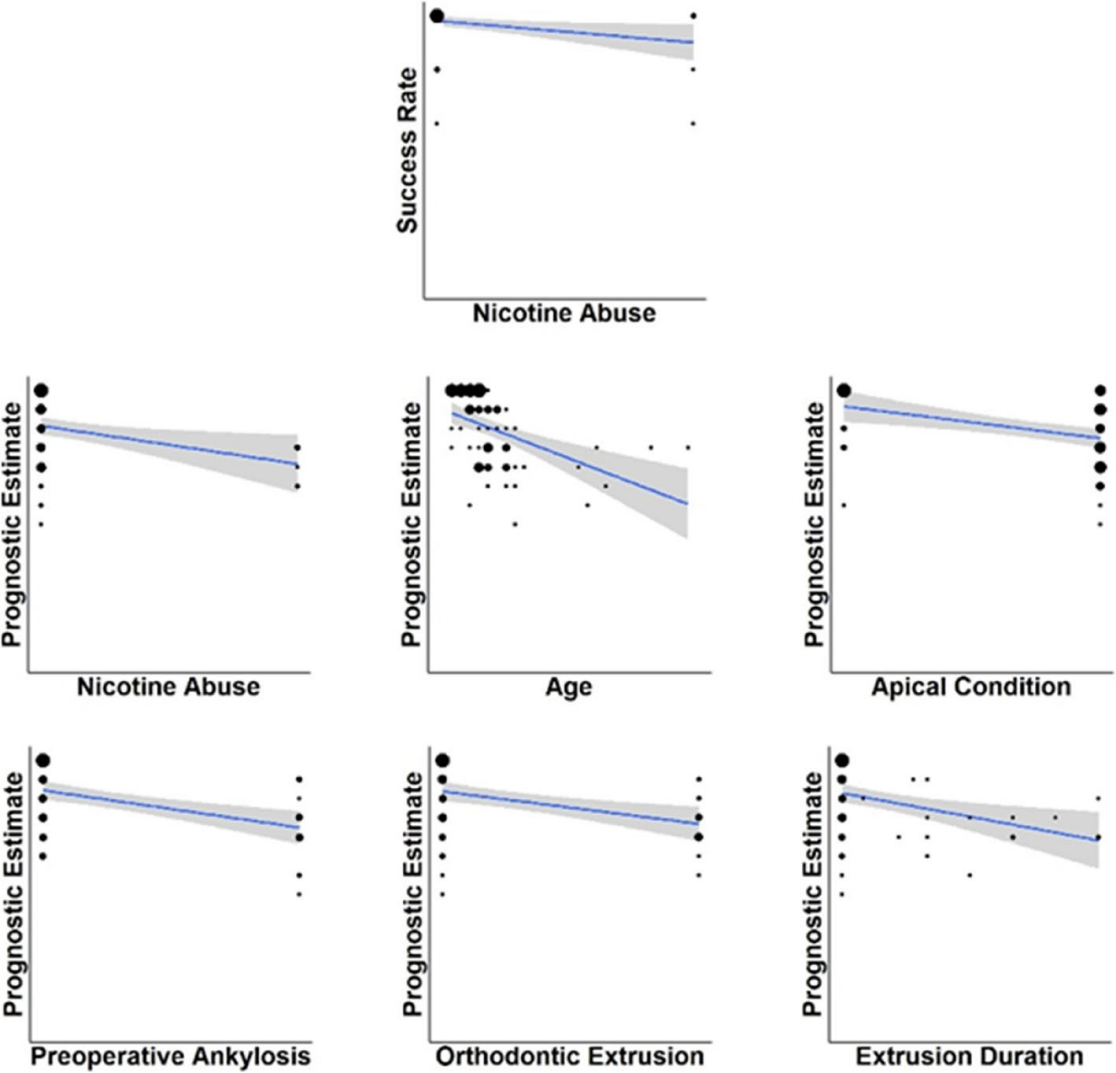
Schematic representation of the regression analysis between the significant influencing factors and the primary outcome 'Success Rate' as well as the secondary outcome 'Prognostic Estimate'

Detailed information on the linear regression analysis for the success rate is provided in Supplementary Figure S1 and Supplementary Table S1, while a schematic overview is presented in Fig. 3.

### Factors influencing the survival rate

As all groups showed a 100% survival rate, no statistical correlation was observed.

### Factors influencing the prognostic estimate

The prognostic estimate was unaffected by adjunctive antibiotic therapy group, sex, displacement severity, jaw location, EST, or intraoperative complications (all *p* > 0.05). Significant predictors included smoking, age, apex condition, preoperative ankylosis, orthodontic extrusion, and extrusion duration.

Smoking reduced the prognostic estimate (β = –14.15; *p* = 0.014; ηP^2^ = 0.089; adjusted R^2^ = 0.075).

Age had the strongest influence (β = –1.30 per year; *p* < 0.001; ηP^2^ = 0.222; adjusted R^2^ = 0.210).

Closed apex reduced prognosis (β = –11.84; *p* < 0.001; ηP^2^ = 0.155; adjusted R^2^ = 0.142).

Preoperative ankylosis had a similar effect (β = –13.80; *p* < 0.001; ηP^2^ = 0.188; adjusted R^2^ = 0.175).

Orthodontic extrusion (β = –12.04; *p* = 0.002; ηP^2^ = 0.143; adjusted R^2^ = 0.130) and extrusion duration (β = –0.49 per month; *p* = 0.003; ηP^2^ = 0.125; adjusted R^2^ = 0.111) were also significant negative predictors.

Detailed results of the linear regression analysis of the prognostic estimate are shown in Supplementary Figures S2–S7 and Tables S2–S7, whereas a schematic overview is provided in Fig. 3.

## Discussion

The present study evaluated the influence of three adjunctive antibiotic therapy regimens and patient-specific factors on the success, survival, and prognosis of autogenous canine transplants over two years. Short-term findings on early healing have been published previously [25].

Current evidence offers no clear guidance on antibiotic therapy in autotransplantation [67, 68]. In related oral surgical procedures, systematic reviews either discourage routine systemic prophylaxis—such as in the replantation of avulsed teeth [24] —or report inconsistent results for dental implants [69]. Considering the predominance of adolescent patients and the potential adverse effects of antibiotics [70], their use warrants critical appraisal within the framework of antibiotic stewardship [71], which aims to preserve therapeutic efficacy and limit resistance.

Because the ethics committee raised concerns regarding the withholding of adjunctive antibiotic therapy through randomization, allocation of antibiotic regimens was instead determined intraoperatively according to extraoral storage time (EST), a well-established prognostic factor in both replantation and transplantation. All ESTs remained below 12 min, far below the critical 60 min threshold for maintaining periodontal ligament viability [31, 32]. However, this approach inherently links EST and systemic adjunctive antibiotic therapy administration and therefore does not allow an independent assessment of either factor. Consequently, our findings should be interpreted as evaluating outcomes under an EST-guided antibiotic allocation strategy. This design precluded a fully independent assessment of systemic adjunctive antibiotic therapy effects, but the inclusion of patients across a broad age range and various root development stages—some with ankylosis—enhances external validity for clinical application.

The overall success rate of 90% and a 100% survival rate confirm the favourable medium-term outcomes reported in the literature, where success rates range from 88 to 94% [72, 73]. While some variability exists across tooth types, canine transplantation poses unique challenges: long, curved roots, complex impaction sites, advanced root formation limiting revascularization, and technically demanding socket preparation [74–77]. These factors may explain slightly lower success rates compared with simpler procedures such as third molar transplantation. The prognostic estimate introduced here reflects cumulative clinical and radiographic stability beyond basic success or survival. No comparable metric has been proposed in previous studies; thus, the favourable mean value of 86% cannot yet be benchmarked. Nevertheless, it provides a practical tool for estimating long-term functional and aesthetic outcomes. High patient satisfaction (mean 1.4) underlines the clinical relevance and acceptability of canine autotransplantation [78, 79].

No association between outcomes and adjunctive antibiotic therapy regimen or EST was identified, consistent with earlier findings on early healing [25]. These data support the assumption that, within a limited EST window, adjunctive antibiotic therapy offers no measurable advantage. This reinforces the rationale for antibiotic stewardship and individualized decision-making based on case-specific risk rather than routine prescription. It should be noted that all groups—including the group without systemic adjunctive antibiotic therapy—were exposed to a doxycycline-containing extraoral storage solution. Therefore, the present findings reflect differences in systemic antibiotic therapy on top of a uniform local antibiotic exposure and should be interpreted accordingly.

Smoking emerged as the only significant predictor of reduced success. The deleterious impact of nicotine on wound healing and bone metabolism is well established [80–82]. Peripheral vasoconstriction, impaired perfusion, and smoke-induced inflammation likely contributed to compromised periodontal repair, as previously shown for early healing in the same cohort [25].

For the prognostic estimate, significant influences included smoking, age, apex condition, preoperative ankylosis, orthodontic extrusion, and extrusion duration. Increasing age correlated with a steady decline in prognosis, in line with reports linking reduced vascularity and cellular regeneration to diminished healing capacity in older individuals [83–86]. Fully developed bone and closed apices demand more invasive surgical access and limit pulp revascularization, increasing the risk of necrosis and subsequent endodontic intervention [87–89]. Similarly, preoperative ankylosis represents a major obstacle, as surgical removal of ankylosed teeth entails extensive osteotomy and inevitable disruption of periodontal ligament integrity [90–92]. This predisposes to resorption or re-ankylosis, explaining the strong correlation with unfavourable outcomes. Failed orthodontic extrusion and its duration also negatively affected prognosis. Chronic mechanical loading and prolonged inflammation during unsuccessful traction attempts can damage periodontal and pulpal tissues [93–95]. These changes may compromise subsequent transplantation and lead to functional or aesthetic shortcomings, such as gingival recession. In addition, extended orthodontic treatment can induce microtrauma and reactive ankylosis, further reducing long-term predictability [13, 96].

Together, these findings emphasize the central role of patient- and tooth-related factors rather than procedural parameters such as antibiotic therapy or minor variations in EST. Clinically, careful case selection and precise surgical timing remain critical—ideally before complete apical closure but after sufficient root development to allow stable handling. Surgeon experience also plays a decisive role in minimizing trauma and optimizing graft adaptation.

The present results support the assumption that adjunctive antibiotic therapy may not be required for successful autotransplantation when strict asepsis and atraumatic technique are ensured. This aligns with current trends in periodontology and oral surgery favoring targeted, evidence-based antibiotic therapy. Nevertheless, the decision should consider systemic conditions, immune status, and intraoperative contamination risk.

Several limitations must be acknowledged. The study’s exploratory, single-centre design and moderate sample size limit generalizability. Allocation based on EST rather than true randomization may introduce bias, although this approach was ethically justified. In addition, variability in relevant biological factors such as apex condition, preoperative ankylosis, and displacement severity across the EST-based groups may contribute to selection bias. Multicentre trials with larger cohorts and stratified designs are warranted to confirm these findings and refine prognostic modelling [97, 98]. The moderate sample size and resulting wide confidence intervals, particularly in Group 2, indicate limited statistical power to detect small intergroup differences; therefore, subtle but potentially clinically relevant effects cannot be excluded and group-specific estimates should be interpreted with caution. In addition, extended follow-up beyond two years could provide insights into long-term root and bone stability, as late complications such as root resorption may emerge over time.

## Conclusions

In conclusion, the present study demonstrates that individualized, rather than routine, adjunctive antibiotic therapy may be preferable for canine autotransplantation, both for initial healing [25] and for long-term success and prognosis. Smoking, advanced age, closed apex, pre-existing ankylosis, and prior orthodontic extrusion attempts—especially of long duration—were identified as key negative prognostic factors. These findings reinforce the importance of comprehensive preoperative risk assessment and patient-specific treatment planning, consistent with the principles of antibiotic stewardship and minimally invasive surgical care.

## Supplementary Information

Supplementary Material 1

Supplementary Material 2

## Data Availability

The datasets used and/or analysed during the current study are available from the corresponding author on reasonable request. Due to the conditions of the ethics approval granted for this project, anonymised raw data cannot be made publicly available. Requests for data access may be submitted to the responsible ethics committee.

## Abbreviations

AS: Apical status
BL: Bone level
CEJ: Cemento–enamel junction
CI: Confidence interval
DO: Dynamic occlusion
DRKS: Deutsches Register Klinischer Studien (German Clinical Trials Register)
EDTA: Ethylenediaminetetraacetic acid
EST: Extraoral storage time
GR: Gingival recession
ICC: Intraclass correlation coefficient
LMU: Ludwig-Maximilians-Universität München
NaCl: Sodium chloride
PD: Probing depth
PC: Proximal contacts
RCT: Root canal treatment
RL: Root length
SD: Standard deviation
SO: Static occlusion
