# Supplementary material for: Autogenous tooth transplantation of canines: a prospective clinical study on the influence of extraoral storage time-guided adjunctive antibiotic therapy and patient-related risk factors affecting success, survival, and prognosis after two years of follow-up
Source: BMC Oral Health. 2026 Jan 31;26:421. doi: 10.1186/s12903-026-07697-w (PMC12955312; doi:10.1186/s12903-026-07697-w)
Supplement: Supplementary file 1 — Supplementary Material 1 [file 12903_2026_7697_MOESM1_ESM.docx]

# **Supplementary Tables**

Coefficients:

Estimate St. Error t-value p-value Eta2 95% CI

(Intercept) 98.033 1.049 93.445 <2e-16 *** - -

Nicotine abuse -8.033 3.506 -2.291 **0.0252** * **0.07** [0.01, 1.00]

**Tab. S1** Coefficients of the linear regression between the influencing parameter 'Nicotine Abuse' and the outcome parameter 'Success Rate'.

Coefficients:

Estimate St. Error t-value p-value Eta2 95% CI

(Intercept) 86.768 1.684 51.511 <2e-16 *** - -

Nicotine abuse -14.149 5.629 -2.514 **0.0144** * **0.09** [0.01, 1.00]

**Tab. S2** Coefficients of the linear regression between the influencing parameter 'Nicotine Abuse' and the outcome parameter 'Prognostic Estimate'.

Coefficients:

Estimate St. Error t-value p-value Eta2 95% CI

(Intercept) 105.773 4.939 21.414 < 2e-16 *** - -

Age -1.300 0.302 -4.303 **5.78e-05** *** **0.22** [0.09, 1.00]

**Tab. S3** Coefficients of the linear regression between the influencing parameter 'Age' and the outcome parameter 'Prognostic Estimate'.

Coefficients:

Estimate St. Error t-value p-value Eta2 95% CI

(Intercept) 93.985 2.907 32.333 < 2e-16 *** - -

Apical condition -11.842 3.434 -3.448 **0.000995** *** **0.15** [0.04, 1.00]

**Tab. S4** Coefficients of the linear regression between the influencing parameter 'Apical Condition' and the outcome parameter 'Prognostic Estimate'.

Coefficients:

Estimate St. Error t-value p-value Eta2 95% CI

(Intercept) 88.796 1.739 51.059 < 2e-16 *** - -

Preop. ankylosis -13.796 3.559 -3.876 **0.000249** *** **0.19** [0.06, 1.00]

**Tab. S5** Coefficients of the linear regression between the influencing parameter 'Preoperative Ankylosis' and the outcome parameter 'Prognostic Estimate'.

Coefficients:

Estimate St. Error t-value p-value Eta2 95% CI

(Intercept) 88.375 1.786 49.470 < 2e-16 *** - -

Orthod. extrusion -12.036 3.656 -3.292 **0.00161** ** **0.14** [0.04, 1.00]

**Tab. S6** Coefficients of the linear regression between the influencing parameter 'Orthodontic Extrusion' and the outcome parameter 'Prognostic Estimate'.

Coefficients:

Estimate St. Error t-value p-value Eta2 95% CI

(Intercept) 87.8015 1.7470 50.258 < 2e-16 *** - -

Extrusion duration -0.4893 0.1608 -3.044 **0.00337** ** **0.12** [0.03, 1.00]

**Tab. S7** Coefficients of the linear regression between the influencing parameter 'Extrusion Duration' and the outcome parameter 'Prognostic Estimate'.
