# Supplementary material for: Autogenous tooth transplantation of canines: a prospective clinical study on the influence of extraoral storage time-guided adjunctive antibiotic therapy and patient-related risk factors affecting success, survival, and prognosis after two years of follow-up
Source: BMC Oral Health. 2026 Jan 31;26:421. doi: 10.1186/s12903-026-07697-w (PMC12955312; doi:10.1186/s12903-026-07697-w)
Supplement: Supplementary file 2 — Supplementary Material 2 [file 12903_2026_7697_MOESM2_ESM.docx]

# **Supplementary Figures**

**
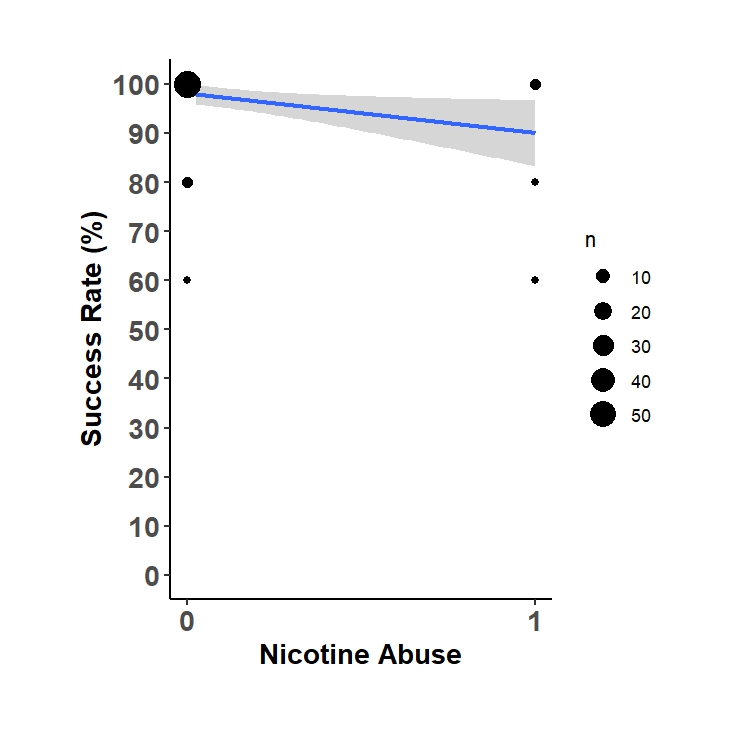
Fig. S1** Linear regression between the influencing parameter 'Nicotine Abuse' and the outcome parameter 'Success Rate'. The size of the points represents the number of data entries. On the x-axis, a value of '0' indicates 'absent' and a value of '1' indicates 'present'.

**
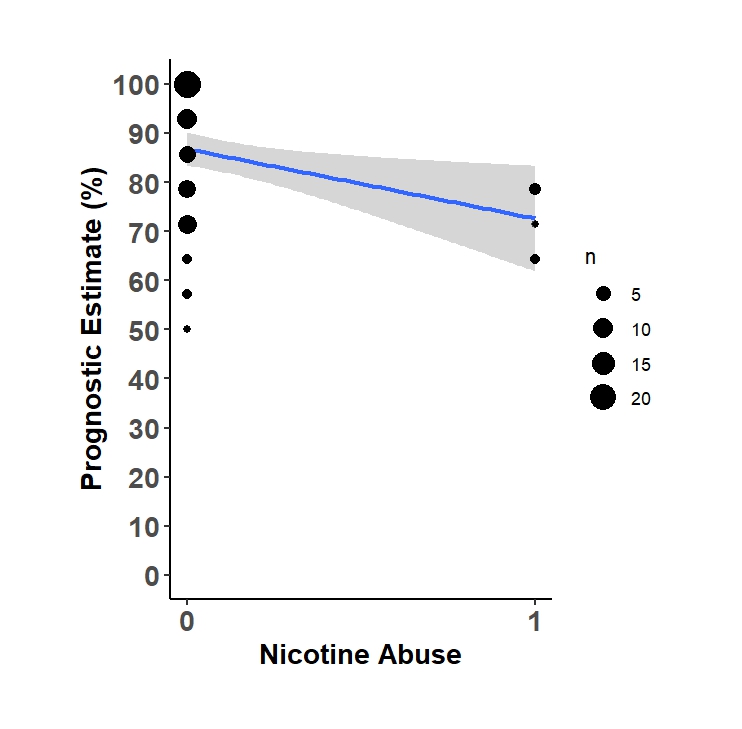
Fig. S2** Linear regression between the influencing parameter 'Nicotine Abuse' and the outcome parameter 'Prognostic Estimate'. The size of the points represents the number of data entries. On the x-axis, a value of '0' indicates 'absent' and a value of '1' indicates 'present'.


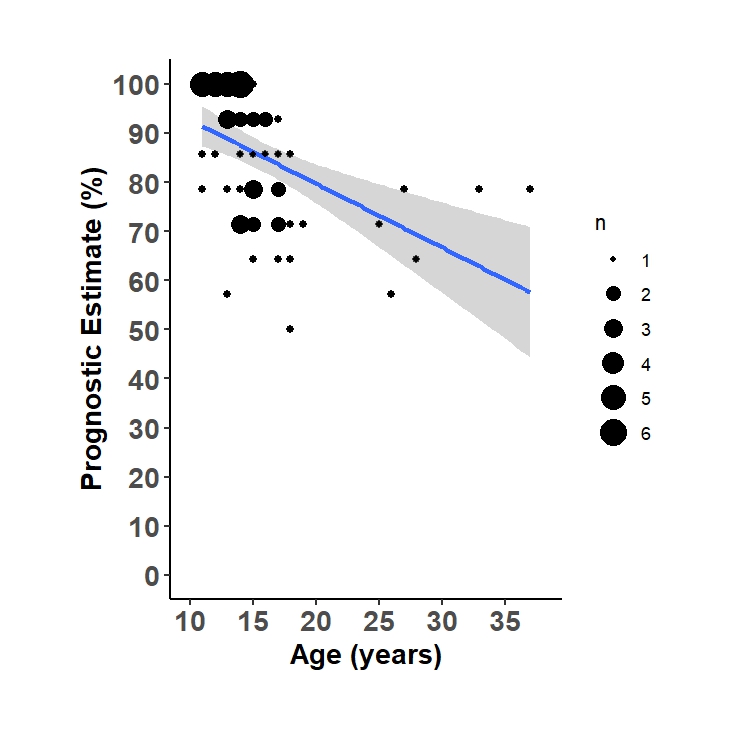
**Fig. S3** Linear regression between the influencing parameter 'Age' and the outcome parameter 'Prognostic Estimate'. The size of the points represents the number of data entries.


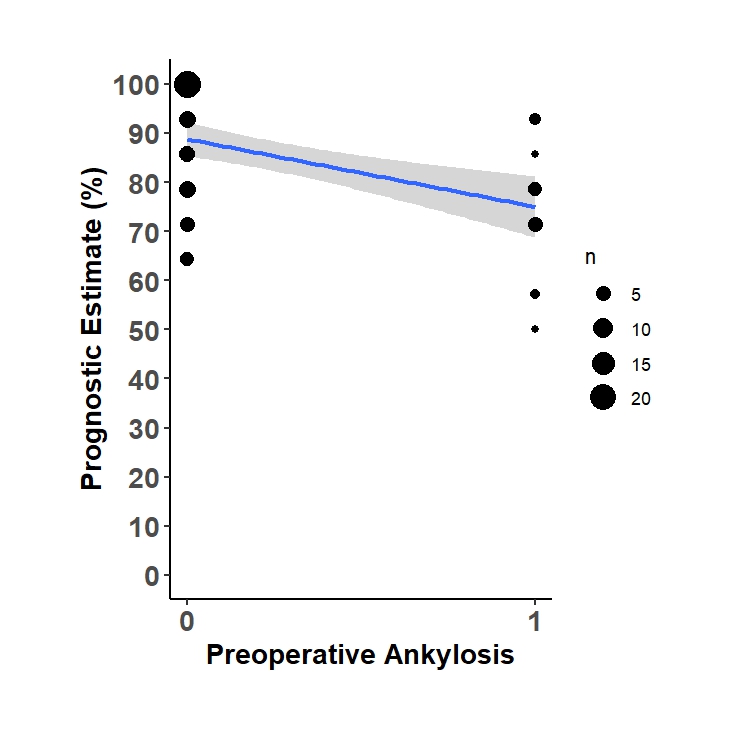

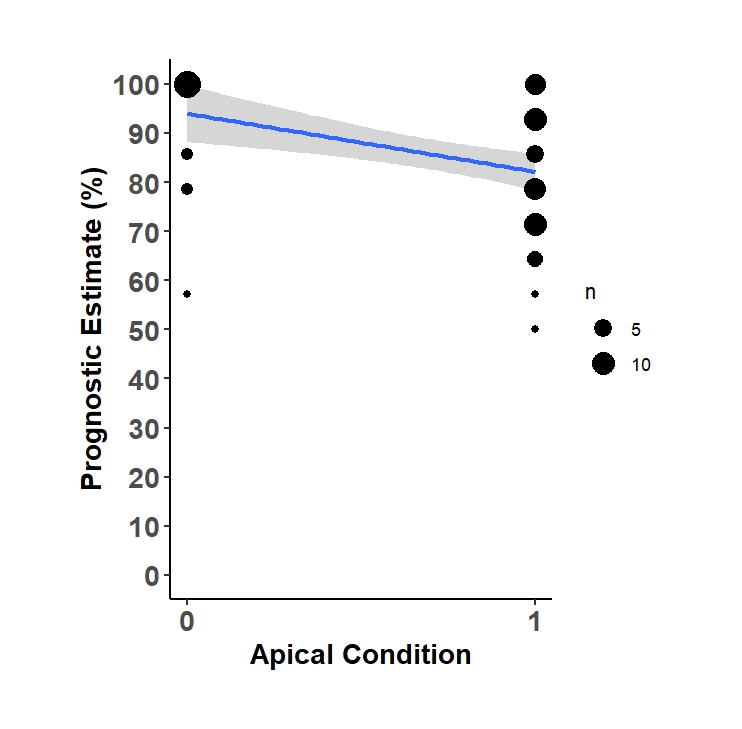
**Fig. S4** Linear regression between the influencing parameter 'Apical Condition' and the outcome parameter 'Prognostic Estimate'. The size of the points represents the number of data entries. On the x-axis, a value of '0' indicates 'open' and a value of '1' indicates 'closed'.

**Fig. S5** Linear regression between the influencing parameter 'Preoperative Ankylosis' and the outcome parameter 'Prognostic Estimate'. The size of the points represents the number of data entries. On the x-axis, a value of '0' indicates 'absent' and a value of '1' indicates 'present'.


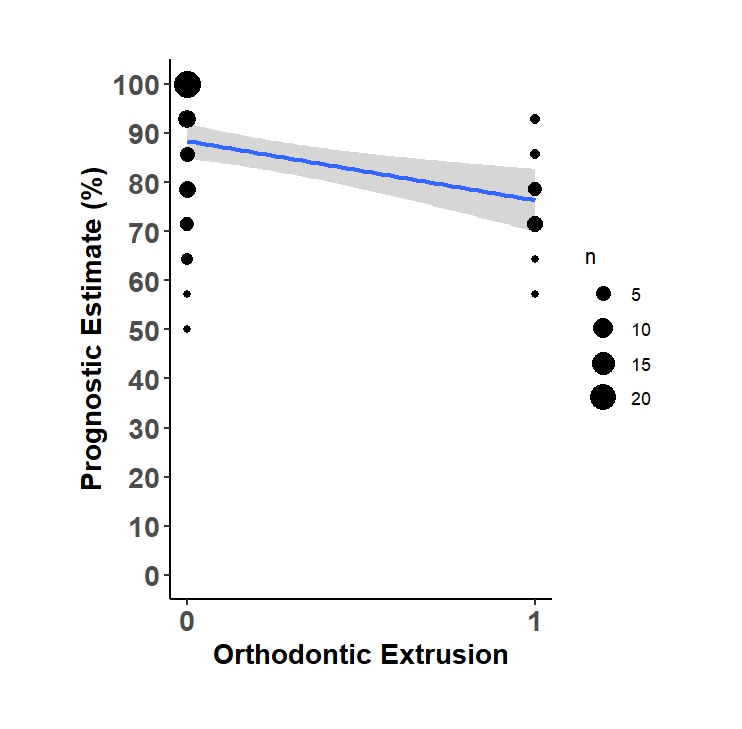
**Fig. S6** Linear regression between the influencing parameter 'Orthodontic Extrusion' and the outcome parameter 'Prognostic Estimate'. The size of the points represents the number of data entries. On the x-axis, a value of '0' indicates 'absent' and a value of '1' indicates 'present'.


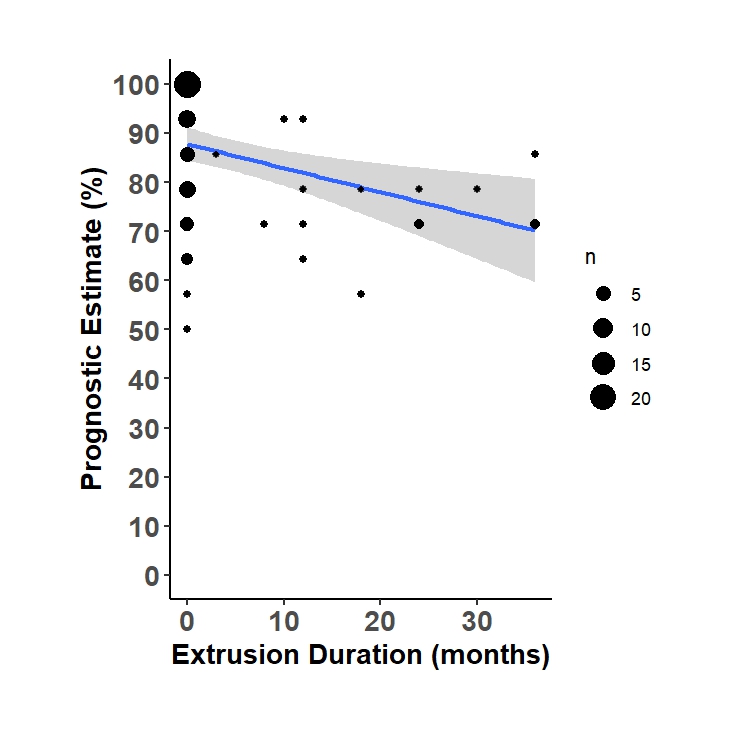
**Fig. S7** Linear regression between the influencing parameter 'Extrusion Duration' and the outcome parameter 'Prognostic Estimate'. The size of the points represents the number of data entries.
